# The ecology of the banded civet (*Hemigalus derbyanus*) in Southeast Asia with implications for mesopredator release, zoonotic diseases, and conservation

**DOI:** 10.1002/ece3.8852

**Published:** 2022-04-28

**Authors:** Ashlea Dunn, Zachary Amir, Henri Decoeur, Bastien Dehaudt, Ilyas Nursamsi, Calebe Mendes, Jonathan H. Moore, Pablo Jose Negret, Adia Sovie, Matthew Scott Luskin

**Affiliations:** ^1^ School of Biological Sciences University of Queensland Brisbane Queensland Australia; ^2^ 255310 School of Environmental Sciences University of East Anglia Norwich UK; ^3^ 255310 School of Environmental Science and Engineering Southern University of Science and Technology Shenzhen China; ^4^ Centre for Biodiversity and Conservation Science University of Queensland St. Lucia Queensland Australia

**Keywords:** abundance modeling, camera trapping, deforestation, emerging infectious disease, wildlife

## Abstract

Habitat loss and degradation threaten forest specialist wildlife species, but some generalist mesopredators exploit disturbed areas and human‐derived food, which brings them into closer contact with humans. Mesopredator release is also important for human health for known zoonotic disease reservoirs, such as Asian civets (Viverridae family), since this group includes the intermediator species for the SARS‐CoV‐1 outbreak. Here we use camera trapping to evaluate the habitat associations of the widespread banded civet (*Hemigalus derbyanus*) across its range in Southeast Asia. At the regional scale, banded civet detections among published studies were positively associated with forest cover and negatively associated with human population. At the local scale (within a landscape), hierarchical modeling of new camera trapping showed that abundance was negatively associated with forest loss and positively associated with distance to rivers. These results do not support mesopredator release and suggest a low likelihood overlap with humans in degraded habitats and, therefore, a low risk of zoonotic disease transmission from this species in the wild. We also estimate that banded civet distribution has contracted to under 21% of its currently recognized IUCN Red List range, only 12% of which falls within protected areas, and a precipitous recent decline in population size. Accordingly, we suggest the banded civet's Red List status should be re‐evaluated in light of our findings.

## Graphical abstract

Habitat loss and degradation generally threaten forest specialist wildlife species, but some generalist mesopredators exploit disturbed areas and human‐derived food, which brings them into closer contact with humans. Understanding mesopredator habitat associations is also important for human health for known zoonotic disease reservoirs, such as Asian civets (Viverridae family), since this group includes the intermediator species for the SARS‐CoV‐1 outbreak. Here we use camera trapping to evaluate the habitat associations of the widespread banded civet (*Hemigalus derbyanus*) across its range in Southeast Asia.

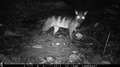

## 1 INTRODUCTION

Understanding the ecology of wildlife hosts of zoonotic diseases (ZD) is a priority for global health monitoring programs (Olival et al., 2017). Fragmentation and other land use changes can increase contact between animals and humans and increase the risk of ZD transmission (Gibb et al., 2020). This is especially true for generalist omnivores and mesopredators whose populations sometimes increase in degraded areas (Filgueiras et al., 2021; Prugh et al., 2009) and are also associated with higher potential ZD burdens (Gibb et al., 2020; Werner & Nunn, 2020). The increase in some medium‐sized carnivores in degraded habitats, sometimes termed “mesopredator release”, could be driven by beneficial habitat, food sources, reduced predation, and competition with apex predators (Prugh et al., 2009). For example, numerous zoonotic disease‐carrying forest‐dependent species benefit from anthropogenic resource subsidies at the edges (Gibb et al., 2020; Luskin, 2010), including mesopredators in Southeast Asia such as leopard cats (*Prionailurus bengalensis*) and common palm civets (*Paradoxurus hermaphroditus*), because they forage on fallen fruit and rodent prey available in oil palm plantations (Chua et al., 2016; Dehaudt et al., 2022; Luskin et al., 2017; Nakashima et al., 2013; Silmi et al., 2021). The release of Asian civets (Family: Viverridae) is especially important because they host numerous ZDs and were the most probable source of the 2003 outbreak of SARS‐CoV and may host SARS‐CoV‐2 (COVID‐19; Guan et al., 2003; Li et al., 2006; Li, 2008; Olival et al., 2017; Lu et al., 2020; Wu et al., 2005). Further, a recent review suggests civets are part of a subset of tropical forest generalist species that may benefit from forest disturbances and thrive in edges, which are a key interface for human‐wildlife interactions and ZD transmission (Filgueiras et al., 2021; Gibb et al., 2020). There is a clear need to determine which civet species pose the greatest ZD risks by persisting in degraded areas where they likely interact with humans. However, there has been little research focusing on the habitat preferences of individual civet species.

The banded civet, *Hemigalus derbyanus* (Gray, 1837), is a prime candidate for ecological and ZD research because its range overlaps with densely populated areas of Southeast Asia and it has been reported to persist in degraded, managed, and logged forests (Bai et al., 2012; Brodie et al., 2015; Li et al., 2012). Banded civets are a widespread but cryptic carnivore that is poorly researched and occurs on the Malay Peninsula (Thailand and Malaysia), Borneo, Sumatra, and some of the Mentawai islands, at elevations from sea level to 1575 m (Francis, 2008; Holden, 2006; Jennings et al., 2013; McCarthy & Fuller, 2014; Mohd‐Azlan et al., 2019; Phillipps & Phillipps, 2018). Even though there has been little targeted research on the species’ ecology or robust empirical work on its population trends, the high levels of forest loss and degradation across the banded civet's range have led to the classification of the species as Near Threatened by the International Union for Conservation of Nature Red List (“IUCN‐RL” hereafter [Ross et al., 2015]).

Here, we conduct a range‐wide synthesis of banded civet habitat preferences with a focus on factors relevant to the species conservation and the risk of ZD transmission to humans (presence and activity in degraded forests). We collated a database of occurrence records from across Southeast Asia and assessed habitat preferences across different spatial scales. First, we used Maxent to map the banded civet's distribution using presence‐only data. Second, we examined landscape‐level habitat associations at the regional scale, inferred from capture rates reported in published camera trapping studies. Third, we used 20 new camera‐trapping sessions from 10 landscapes across the species’ range to determine local (within‐landscape) habitat associations and behavior. We assessed the species’ relationship with forest size, forest edge, forest integrity (a catch‐all term we use to include direct and indirect effects of forest edges, fragmentation, and logged areas, as defined by Grantham et al., 2020), as well as human densities and night lights. This latter variable is important for assessing the likelihood of human interactions with crepuscular or nocturnal banded civets (Mohd‐Azlan et al., 2019), as night lights are associated with nocturnal human activity. We also aimed to resolve the species elevational preferences, with prior reports suggesting the species was more common at relatively high (>600 m) and low elevations (<200 m) rather than in between (Brodie et al., 2015; Jennings et al., 2013). We also examined the influence of rivers on the local abundance of banded civets, noting previous work suggested riparian areas are unsuitable habitat (Ross et al., 2016). Finally, we were interested in interspecific interactions that could facilitate transmission of ZD between civet species, since there has been more epidemiological research on closely related civets such as masked palm civets (*Paguma larvata*). Temporal overlap can increase the likelihood of interactions. Therefore, we examined the activity pattern overlap for sympatric civet species that may be competitors, as well as overlap of potential prey (rodents) and a potential predator (clouded leopards, *Neofelis nebulosa* & *N*. *diardi*). We also looked at whether banded civets show altered activity patterns near humans, as has been suggested for a wide variety of wildlife species (Gaynor et al., 2018).

## 2 METHODS

### 2.1 Species description

**FIGURE 1:**
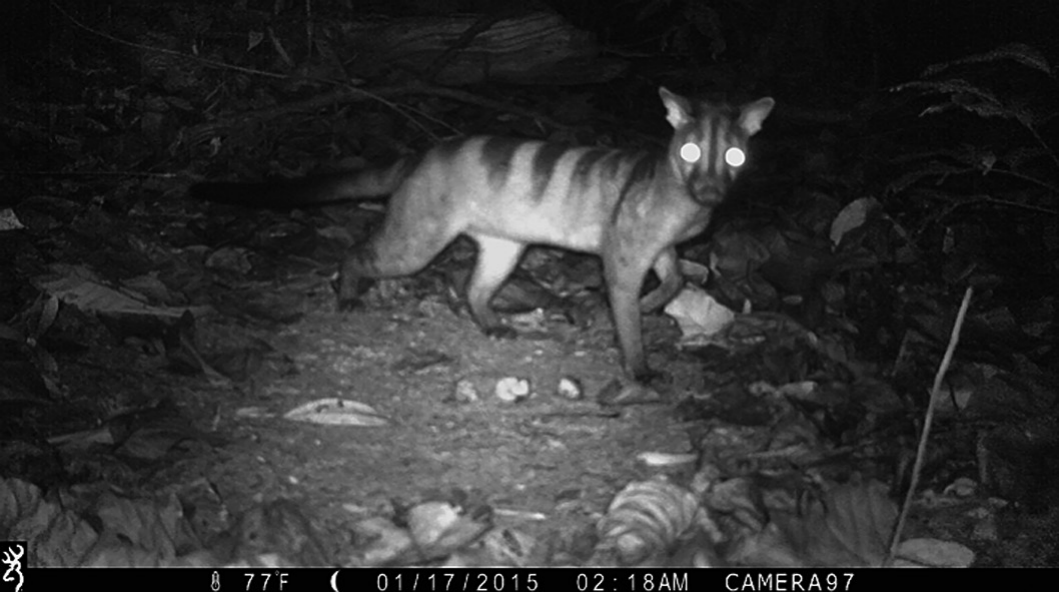
Banded civet camera trap image from Danum Valley, 2020

Banded civets are notable for their pale brown or grey body painted with 7–8 distinctive black vertical bands and stripes along the face (Figure 1) (Jennings et al., 2013; Phillipps & Phillipps, 2018; Veron et al., 2004). The banded civet weighs between 1 and 3 kg, with a head‐body length of 45–46 cm and a tail length of 23–36 cm (Francis, 2008; Jennings et al., 2013; Phillipps & Phillipps, 2018). It is sometimes confused with the smaller banded linsang (*Prionodon linsang*; 600–800 g), as both have elongated and banded bodies (Phillipps & Phillipps, 2018). It is semi‐arboreal and sometimes rests in low tree holes during the day (Brodie & Giordano, 2011; Jennings et al., 2013; Kitamura et al., 2010; Phillipps & Phillipps, 2018). Unlike other civets that are omnivorous and may disperse seeds, banded civets appear to be strict carnivores, with the limited number of diet observations to date being limited to invertebrates and small vertebrates, including earthworms, ants, spiders, frogs, freshwater crabs, rats, and birds (Colon & Sugau, 2012; Francis, 2008; Phillipps & Phillipps, 2018; Ross et al., 2016). The banded civet has several interesting morphological traits, including the presence of sensory hair between the pads of their feet for sensing prey (Phillipps & Phillipps, 2018), strong retractable claws (Francis, 2008), and a tail that swells when threatened (Louwman, 1970).

### 2.2 Data collection

We compiled presence and absence data for the banded civet from four sources: (1) captures reported in published camera‐trapping studies; (2) camera‐level capture histories from new camera‐trapping sessions conducted across 10 landscapes in Southeast Asia; (3) presence‐only data from the Global Biodiversity Information Facility database (GBIF, 2019), a global repository of biodiversity data including museum records and citizen science reports; and (4) presence‐only data from the Borneo Carnivore Database (Ross et al., 2016). The camera trapping datasets and analyses are part of the Ecological Cascades Lab standardized approach to multi‐scale species‐specific analyses (i.e., replicated from Dehaudt et al., 2022, based on Ke & Luskin, 2019). Presence data consist of georeferenced occurrence records, defined as the coordinates of a location where the species was observed. We defined a camera‐trapping study as a continuous sampling effort using at least 5 cameras within a landscape (10–1000 km^2^). We refer to the sampling area as a “landscape”, which was usually a national park, a production forest, or a collection of forest patches within a 100 km^2^ area. We collated camera‐trapping data from 49 landscapes, including 20 new camera‐trapping sessions at 10 landscapes (Figure 1; Table 2).

### 2.3 Collating published camera trapping records for regional analyses

We compiled published camera‐trap records by searching Web of Science with the following criteria: “camera trap*” AND Asia* or Thai* or Malay* or Indonesia* or Singapore* or Cambodia* or Vietnam* or Lao* or Myanmar* or Burm* or Sumatra* or Borne*. We selected from the list of returned studies those that were written in English and reported relevant results for the species of interest, including sampling effort (number of cameras, and deployment length or total trap nights), and number of independent captures (generally defined based on a 30–60 min interval between captures of the same species, referred to as “independence period”). We examined the references listed in key papers to identify and include further sources. We included all tropical forest camera trapping studies that used unbaited cameras placed <0.4 m height, usually facing trails or other areas determined by researchers to be used by wildlife. This is the standard deployment approach used in the region and suitable for the majority of semi‐terrestrial species >1 kg (Rovero & Ahumada, 2017). From each study, we recorded the location (forest name and coordinates), capture and effort data, and a variety of other covariates available (Table S2). We grouped multiple studies from the same landscape in a given year by summing captures and effort among the studies and averaging the covariate values.

### 2.4 New camera‐trapping sessions

**FIGURE 2:**
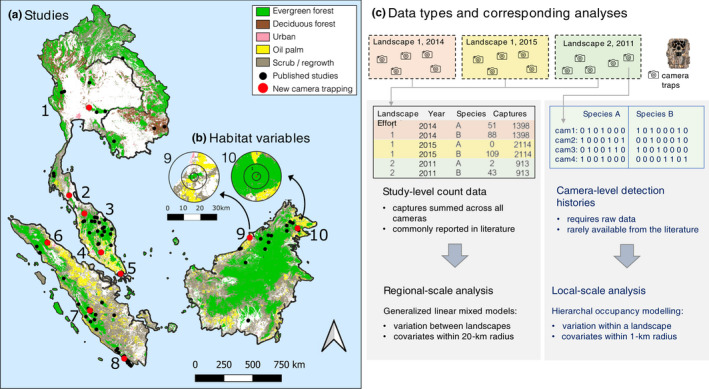
Study area and diagram of camera trapping data types and analyses. (a) Landscapes where camera trapping was undertaken, with black circles showing the location of published camera trapping studies and red circles showing locations of new camera trapping sessions conducted by the Ecological Cascades Lab program (“ECL” hereafter), including Pasoh data from the Tropical Ecology Assessment and Monitoring (“TEAM”) Network. Note that ten landscapes were surveyed, including Khao Yai in Thailand (outside of the IUCN‐RL range) since there was little authoritative information about the species northern range limits, but since the species was not detected, we excluded from the occupancy analyses. The map inset (b) shows the process of extracting habitat covariates, which were averaged for 20‐km radius around all landscape‐level surveys and for the ECL datasets, these covariates were averaged for the 1‐km radius around each camera. The left side of panel (c) shows the structure of the study‐level species counts per landscape that was analyzed using Poisson GLMMs, where the “landscape” was the sampling unit. The right side of panel (s) shows the camera‐level capture histories that were used in hierarchical abundance modeling. Panel (c) summarizes the data flow from the landscape‐level captures reported by published studies used in GLMMs versus the camera‐level detection histories used in the abundance modeling

We conducted 20 new camera‐trapping sessions in 10 lowland and hill dipterocarp forest in Thailand, Peninsular Malaysia, Singapore, Sumatra, and Borneo between December 2013 and March 2019. We deployed between 18 and 78 passive infrared camera traps across sampling areas ranging from 10 to 813 km^2^ (Figure 2). We standardized deployment methods across all landscapes (see Tables S1–S3 for landscape characteristics, variable descriptions, sampling effort, capture rates, and naive occupancy). Cameras were placed within a pre‐mapped grid and spaced at least 500 m apart in large, forested landscapes (>50 km^2^) and 100–500 m apart in smaller forest patches. Cameras were attached to trees 0.3 m above ground and placed along nearby hiking trails or natural wildlife trails and deployed for 60–90 days. In order to ensure that model outputs were spatially comparable across multiple landscapes and to prevent spatial pseudo‐replication, we resampled the capture data into hexagonal grid cells with a short diagonal of 1 km (0.87 km^2^ per cell) following Rayan and Linkie (2020). In most cases, each sampling unit contained only one camera associated with a unique value for each habitat covariate, but we averaged covariate values when multiple cameras fell within the same grid cell (Table S1). We considered captures independent if they occurred at least 30 min apart. We produced detection history matrices based on a sampling occasion of three days, and containing presence/absence data (0 = species not detected; 1 = species detected; NA = inactive sampling unit or occasion). We note that we did not sample peat swamp forests or freshwater swamp forests or mangroves.

### 2.5 Mapping distribution and probability of presence

First, to assess if the IUCN‐RL species range accurately captured the species current distribution and infer its recent range and population contraction, and given species is forest‐dependent, we used QGIS to clip the current IUCN “extent of occurrence” (EOO) distribution map by removing the areas that were not forest based on a detailed remote sensing habitat layer (IUCN, 2020; Miettinen et al., 2016). Some may considered this to be determining the Area of Habitat (AOH) of the species (Brooks et al., 2019). We also calculated the percentage of the AOH forest that is protected, based on the IUCN World Database on Protected Areas (UNEP‐WCMC & IUCN, 2021).

We mapped banded civet habitat suitability using Maxent (Phillips et al., 2006), based on presence‐only data and 8 spatial layers (Table S2). We used the combined dataset of occurrence records but removed records dating from before the year 2000, in order to avoid including false positives in areas where the species may no longer be present. To minimize sampling bias, we included a “bias file” that accounts for sampling effort and we limited the spatial extent to reduce pseudo‐absences, as suggested by Fourcade et al. (2014) and Stolar and Nielsen (2015). To ensure proper fit the Maxent AUC (Area Under the Curve) value was assessed after incorporating “bias file”, which improved the AUC, and we tested model performance using receiver operating characteristic (ROC) analysis, setting aside 15% of the data (Fourcade et al., 2014; Kramer‐Schadt et al., 2013).

**FIGURE 3:**
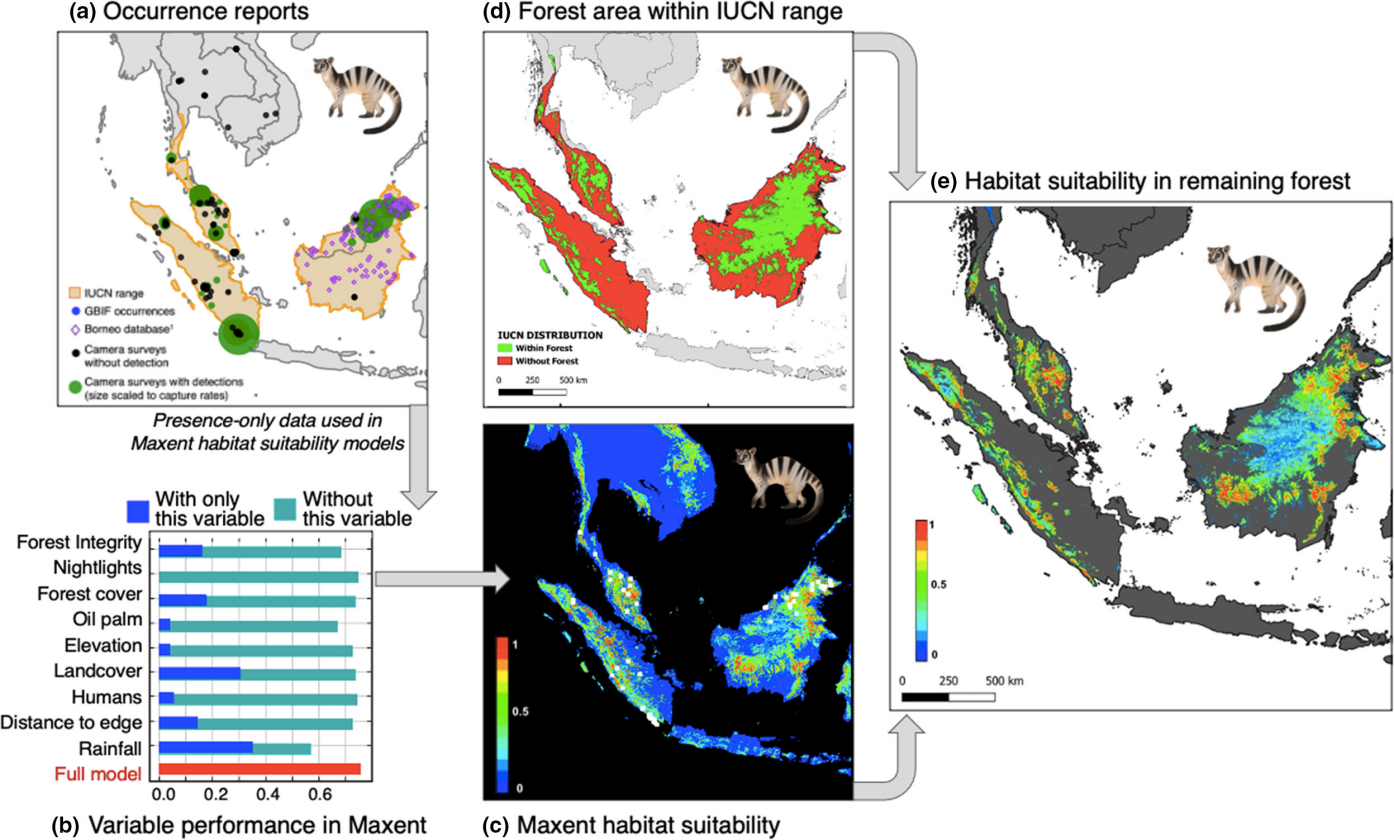
The banded civet range, habitat associations, and habitat suitability within remaining forest. In panel (a), the shaded area shows the IUCN Red List range (extent of occurrence or “EOO”) and the location of occurrence records, colored by data source. Panel (b) shows the Jackknife graph of variable performance in the Maxent habitat suitability modeling using the regularized training gain. The dark blue bars show the predictive power of a model using only the denoted variable, while the teal bars show the predictive power of the full model except the denoted variable, the latter highlighting whether the variable captures unique information. Panel (c) shows the predicted probability of presence throughout Southeast Asia, including non‐forested areas. Panel (d) shows forest cover within the species range as of 2015 with non‐forested areas assumed to be unoccupied. Panel (e) shows the predicted probability of presence within remaining forest

Our environmental layers included biogeographical factors (elevation, landscape cover, mean annual rainfall, forest cover, forest landscape integrity index (FLII)), as well as anthropogenic factors (human population density, nightlights, and oil palm cover) (Table S2). We report the Jackknife training gain test results to show the relative contribution of each predictor to the model. We follow the Maxent guidelines for mapping using the transformed complementary cloglog output, which provides scaled probability of presence between zero and one. We generated maps in QGIS, including the Maxent model output layer clipped to show probability of presence in remaining forest within the AOH (i.e., within the species’ EOO; Figure 3).

### 2.6 Assessing regional habitat associations with GLMMs

We investigated relationships between detection rates from published and new camera‐trapping studies and landscape‐level environmental and anthropological factors using generalized linear mixed models (GLMMs). We treated detections as count data and used a zero‐inflated Poisson distribution (ZIP), and included study effort (measured in trap nights) as a fixed effect and landscape as a random effect (factor with 49 levels). We choose to use the raw count data as opposed to a relative abundance index (RAI, usually independent photos per 100 trap nights) following Ash et al. (2020), and we note that these approaches do not account for variation in detection probability and thus do not linearly reflect true abundance (Sollmann et al., 2013). Therefore, in this analysis, we are implicitly assuming that detection probability does not vary between studies and acknowledge this may introduce measurement error. We also acknowledge that there is unexplained variation in captures owing to slight differences in equipment and deployment methodology among studies. Both sources of measurement error may reduce our modeling power and our chances of detecting significant “true” relationships.

We used the ZIP GLMMs to test the effect of eight environment descriptor variables (Table 5) on banded civet relative abundance among landscapes. All covariates were derived from spatial layers and describe the attributes within a 20‐km radius around the centroid of each landscape. Our spatial covariates included forest cover, night light intensity, human population, forest integrity, elevation, forest area (km^2^), and annual precipitation. Sources for spatial layers and the year of their measurement are summarized in Table S2. We used this vast area (1256 km^2^) to account for some large camera trapping grids and the possible low precision of centroid coordinates provided or inferred from the landscape description in some studies. For each variable, we tested a linear and a non‐linear model since other studies have found a variety of Bornean species show non‐linear responses to similar variables (Brodie et al., 2015). We used AICc model selection to identify the most parsimonious model and considered models within 2AIC units of the best model to be competing models (Burnham & Anderson, 2002).

### 2.7 Assessing local‐scale (within‐landscape) habitat associations using Royle‐Nichols abundance modeling

We assessed the effect of habitat variables on banded civet abundance while accounting for imperfect detection using the Royle‐Nichols (RN) hierarchical modeling approach (Royle & Nichols, 2003). The RN model uses presence‐absence data to infer abundance per sampling unit (*lambda*) by exploiting the positive relationship between heterogeneity in individual detection probability (*r*) and the species’ abundance, where predicted values from these models are assumed to scale linearly with true abundance. We incorporated covariates to model heterogeneity in abundance (*lambda*) using a log‐link function, and to model heterogeneity in individual detection probability (*r*) using a logit‐link function (Royle & Nichols, 2003). Our reduced model included camera trapping sessions as a categorical fixed effect in abundance parameter (*lambda*) to account for differing abundance per camera trapping session and included the effort per sampling unit (in trap nights) as a continuous fixed effect in the detection parameter (*r*) to account for when multiple cameras were grouped into a single 0.87 km^2^ hexagon sampling unit. We did not include any additional covariates beyond effort per sampling unit in the detection parameter (*r*) because we assumed detection probability was constant across cameras because we placed all of them along human or wildlife trails in similar lowland tropical forest habitats. We built upon our reduced model to assess relationships with banded civet abundance (*lambda*) using variables described previously for the regional‐scale GLMMs, this time calculated within a 1‐km radius around each camera, as well as with additional local‐scale predictors including distance to forest edge, distance to river, and active forest loss within 1 km (Table S2). We filtered out correlated variables (|*r*| > .6), developed univariate and multivariate models, and implemented AICc model selection with reference to the reduced model. We implemented the hierarchical abundance modeling using the “unmarked” package in R (Fiske & Chandler, 2011). Finally, assessed any remaining overdispersion on our most parameterized model by calculating “C‐hat" and “*p*‐value” scores using the Mackenzie‐Bailey goodness‐of‐fit test using 1000 simulations, which was updated to work with RN models in the “AICcmodavg” package (Mackenzie & Bailey, 2004; Mazerolle & Mazerolle, 2017).

### 2.8 Analysis of diel activity patterns

We used time‐stamped detections from our new camera‐trapping sessions to investigate variability in the banded civet's diel activity within the study area. We computed von Mises kernel density estimates and coefficients of temporal overlap with multiple civet species in R. For each species we created a kernel density function which is a smoothed curve observations over time. We used the Schmid and Schmidt (2006) Dhat estimator to compute the coefficient of overlap for each species pair, where we used Dhat_1_ if we generated less than 60 independent detections, and Dhat_4_ if we generated more. To fit the circular kernel density function, we used the “fitact” function. We used the “OverlapEst” function in package “Overlap” (Meredith & Ridout, 2014) to calculate the Dhat coefficient of overlap. A low coefficient of overlap between sympatric populations indicates temporal avoidance (Sovie et al., 2019).

We tested if disturbances including forest edges, forest integrity, and human footprint affect species behavior by testing for significant differences in diel activity patterns (Grantham et al., 2020; Venter et al., 2016). Specifically, we split our time‐stamped detections based on the median value of each disturbance variable, and ran a bootstrap procedure to simulate 1000 distributions of activity pattern data to conduct a Wald test using the function compareAct() in the R package “activity” (Rowcliffe et al., 2014). When significant differences in activity patterns were detected, the coefficient of overlapping was calculated from the R package “overlap” (Ridout & Linkie, 2009).

## 3 RESULTS

### 3.1 Distribution and habitat suitability mapping

The banded civet's IUCN‐RL EOO, that is, the area covered by its known range in the study region is 2,320,382 km^2^ (Figure 3a). Since banded civets are forest‐dependent, we estimated their Area of Habitat (AOH) based on the remaining 2015 forest cover and found this was 66% lower than the EOO (783,820 km^2^) and that only 12% falls within protected areas (Figure 3c, Table 1).

**TABLE 1:** Range, habitat availability, and naïve occupancy of the banded civet in Southeast Asia

Regions	EOO (km^2^)	AOH (km^2^)	EOO that is forested (%)	EOO that is forested and protected (%)
Borneo	734,433	321,603	43.8	6.7
Thai Peninsula	57,324	12,065	21	16.3
Malay Peninsula	130,937	46,030	35.2	12.5
Sumatra	430,037	84,888	19.7	7.6
SE Asia total	1,352,731	464,586	34.3	7.9

Note

EOO refers to the extent of occurrence, which we calculated as the total area within the IUCN‐RL range in each region (km^2^). We updated the EOO based on the forested area in 2015 remaining within the IUCN‐RL EOO (Miettinen et al., 2016), which may be interpreted more correctly as the remaining habitat available (AOH). Protected areas were taken from Protected Planet database (IUCN, 2010). Results per country are available in Table S3.

For our Maxent modeling, we gathered a total of 186 geo‐referenced occurrence records for the banded civet, including 49 from published studies, 8 from new camera‐trapping sessions, 12 from GBIF, and 125 from the Borneo carnivore database (Table 2). Naïve occupancy (the proportion of studies with detections compared to the total number of studies) was about 70% (Table 3). Maxent model performance for the banded civet was high (AUC for the ROC curve on the test data = 0.762) and there was high habitat suitability in the lowlands fringing mountains of Peninsular Malaysia, Sumatra, and Borneo (Figure 3c,e). The variable containing the highest amount of information when used in isolation to model habitat suitability was landscape forest cover (Figure 3b). Forest cover and forest integrity positively influenced probability of presence and there was a humped‐shaped relationship with annual rainfall peaking from 2000 to 4000 mm year^−1^ (Figure S1).

**TABLE 2:** Data sources and sample sizes for the four analyses

Source of presence/absence locations	*N*
Landscapes with CT data for GLMMs (presence & absence)	70
(landscapes with CT presences for Maxent)	41
Borneo carnivore database (presence‐only)	125
GBIF (presence‐only)	12
New CT sessions for RN hierarchical models (landscapes)	20 (10)
(landscapes with presences for Maxent)	8
Detections in new CT sessions for activity patterns	405
Total Presences for Maxent	196

Note

The capture information from the new camera trapping (CT) sessions was included in the Maxent, GLMM analyses, and the activity pattern analyses.

**TABLE 3:** Landscape‐level naïve occupancy of the banded civet from camera trapping

Regions	Landscapes surveyed	Landscapes with detections	Naïve occupancy	Camera stations	Effort (trap nights)	Independent detections	RAI
Borneo	15	13	0.867	1237	62,369	343	0.55
Thai Peninsula	3	3	1	235	26,165	55	0.21
Malay Peninsula	16	8	0.5	1850	136,929	425	0.31
Sumatra	7	5	0.714	1665	103,450	163	0.16
SE Asia Total	41	29	0.707	4987	328,913	986	0.30

Note

The 70 camera trapping studies were grouped into “landscapes” usually national parks or other defined forests separated by a hard border (agriculture, urban areas). Landscapes were considered to be occupied if our study species was ever captured there, regardless of the time of sampling. Some landscapes were sampled on multiple occasions, which is why the total studies available for the GLMM exceed the number of landscapes. We also present results separately for each country in Table S4. RAI is a relative abundance index, calculated as the number of independent captures per 100 trap nights.

### 3.2 Regional‐level occurrence predictors assessed with GLMMs

The top model explaining regional banded civet detections included a positive effect of forest cover (*β* = 1.752, SE = 0.45; Table 4). The human population model was also well supported (ΔAIC = −1.3), with banded civet detections decreasing with human population (*β* = −1.7, SE = 0.44, quadradic term *β* ^2^ = −0.51, SE = 0.22). There was no support for multivariable models.

**TABLE 4:** Model performance for assessing regional variation in camera trap captures

Model	*K*	AICc	ΔAIC	AICwt
Forest cover	6	440.6	0	0.62
Human population^2	7	441.9	1.3	0.32
Forest integrity^2	7	448.4	7.8	0.01
Human population	6	448.4	7.8	0.01
Oil palm^2	7	448.7	8.1	0.01
Roughness^2	7	450.2	9.6	0
Reduced model	5	450.3	9.7	0

Note

Univariate linear and non‐linear model selection from the zero‐inflated Poisson GLMM assessing variation in banded civet independent captures, including study effort as a fixed effect and landscape as random effect. Models that performed worse than the reduced model were not included. All covariates were averaged for the 20‐km radius areas surrounding the study, then centered and standardized so effect sizes can be interpreted relative to each other. Human population was logged prior to scaling. Independent captures are usually defined as photos separated by 20–60 min. The results are conservative with the signal from any significant trends overcoming the variation induced by sampling methodology. All models included the same number of parameters (94 observations from 44 landscapes). Tests of non‐linear relationship are shown with the “^2” notation.

### 3.3 New camera trapping and local‐scale hierarchal abundance modeling

**FIGURE 4:**
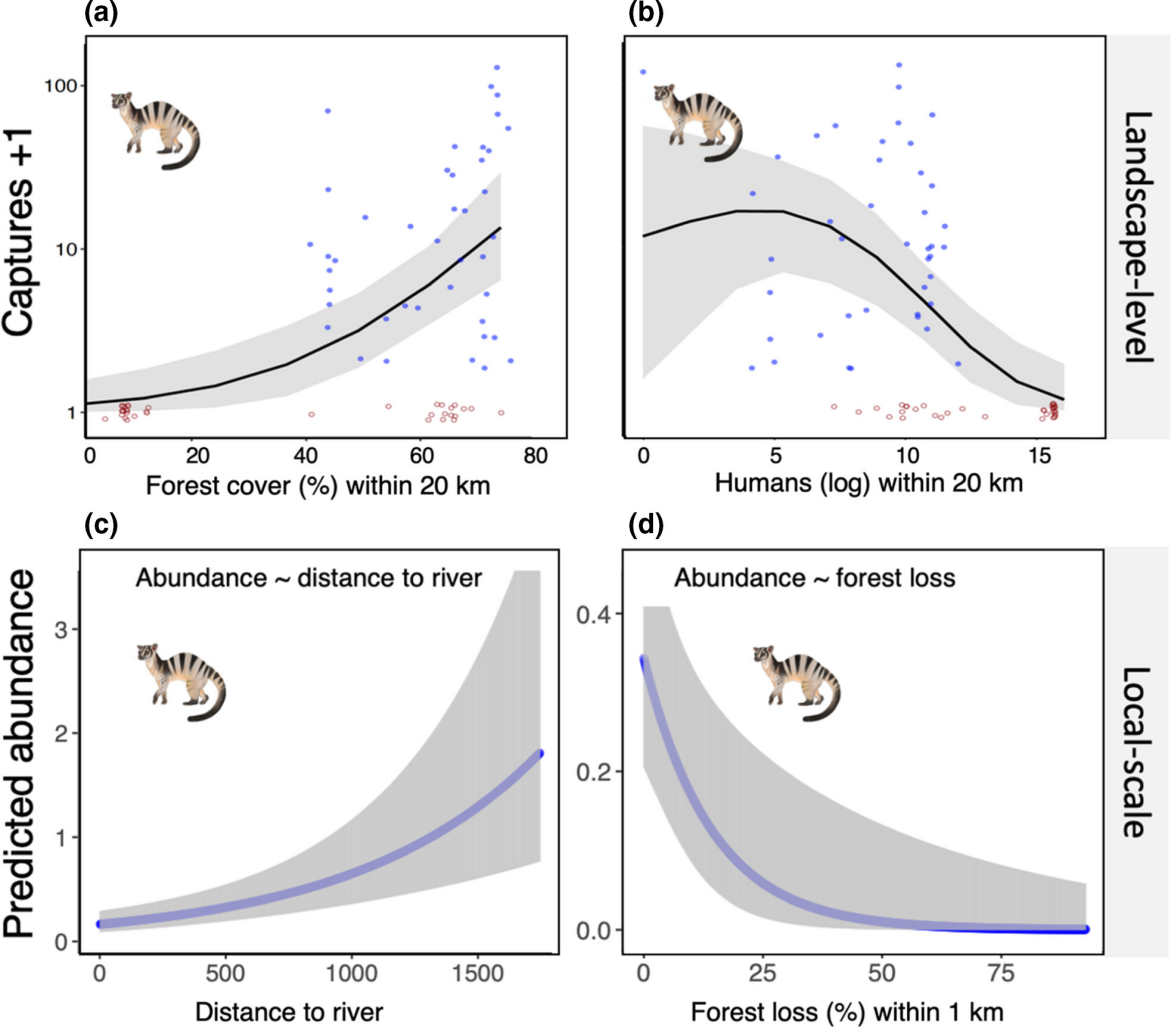
Predictors of regional and local variation in the banded civet. Panels (a) and (b) show regional habitat associations with each data point reflecting the total captures for an entire camera trapping session and the covariates describe the 20‐km radius area covering the study landscape. These relationships were investigated using ZIP GLMMs. Panels (c) and (d) show local habitat associations, where covariates were measured in the 1‐km radius area around each camera. Local‐scale relationships were investigated using RN hierarchical abundance models that account for imperfect detectability. All covariates were centered and standardized prior to modeling, so effect sizes can be compared between variables. Inclusion of the particular covariates was chosen based on AICc model selection (Table 4). Note the linear relationships can appear curved due to the link function. Red points show absences (jittered) while blue points show presences

We obtained 405 independent captures from 8 landscapes (18 sessions, 4,987 cameras, 328,913 trap nights; Table 3). We did not detect the species in Singapore, which is within the species range but reported to have been extirpated in the early 1900s, or in Khao Yai National Park in Thailand, which is just outside the species range. The top RN abundance model included negative associations with rivers (*β* = 0.399, SE = 0.09) and recent forest loss (*β* = −0.919, SE = 0.37; Table 5; Figure 4) and showed excellent fit with no remaining overdispersion (C‐hat = 0.29, *p *= .09). There was only minor support for a positive association with elevation but we note this would only be valid over the relatively low elevation sites sampled.

**TABLE 5:** Model performance for assessing local (within‐landscape) variation in abundance

Model	AIC	*K*	ΔAIC	AIC weight
~ Distance to river + Forest Loss	2860.2	22	0.000	0.7486
~ Distance to river + Elevation + Forest Integrity	2863.0	24	2.787	0.1859
~ Distance to river + Forest Integrity	2865.5	22	5.342	0.0518
~ Distance to river + Elevation	2869.4	22	9.179	0.0076
~ Distance to river	2870.1	21	9.960	0.0051
~ Forest Loss	2880.1	21	19.923	0.00004
~ Elevation	2882.6	21	22.411	0.00001
~ Forest Integrity	2882.7	21	22.481	0.00001
Reduced model	2891.2	20	31.047	0.0000001

Note

We resampled all cameras into 3.46 km^2^ hexagon grids, and fit RN hierarchical abundance models with sampling effort per grid as a covariate affecting detection probability. We included the camera trapping session as a covariate affecting abundance in all models to account for variation between landscapes and between surveys in the same landscape. The table only report the results of our covariate of interest and the direction of the three best covariates effect are shown in Figure 4. Multi‐variate models were only reported if they improved performance by >2 AICc points. Note the reduced model still accounts for lambda (abundance parameter) differing according to each of the landscapes and camera‐trapping sessions and r (detection parameter) is being influenced by the sampling effort at each grid.

### 3.4 Activity patterns

**FIGURE 5:**
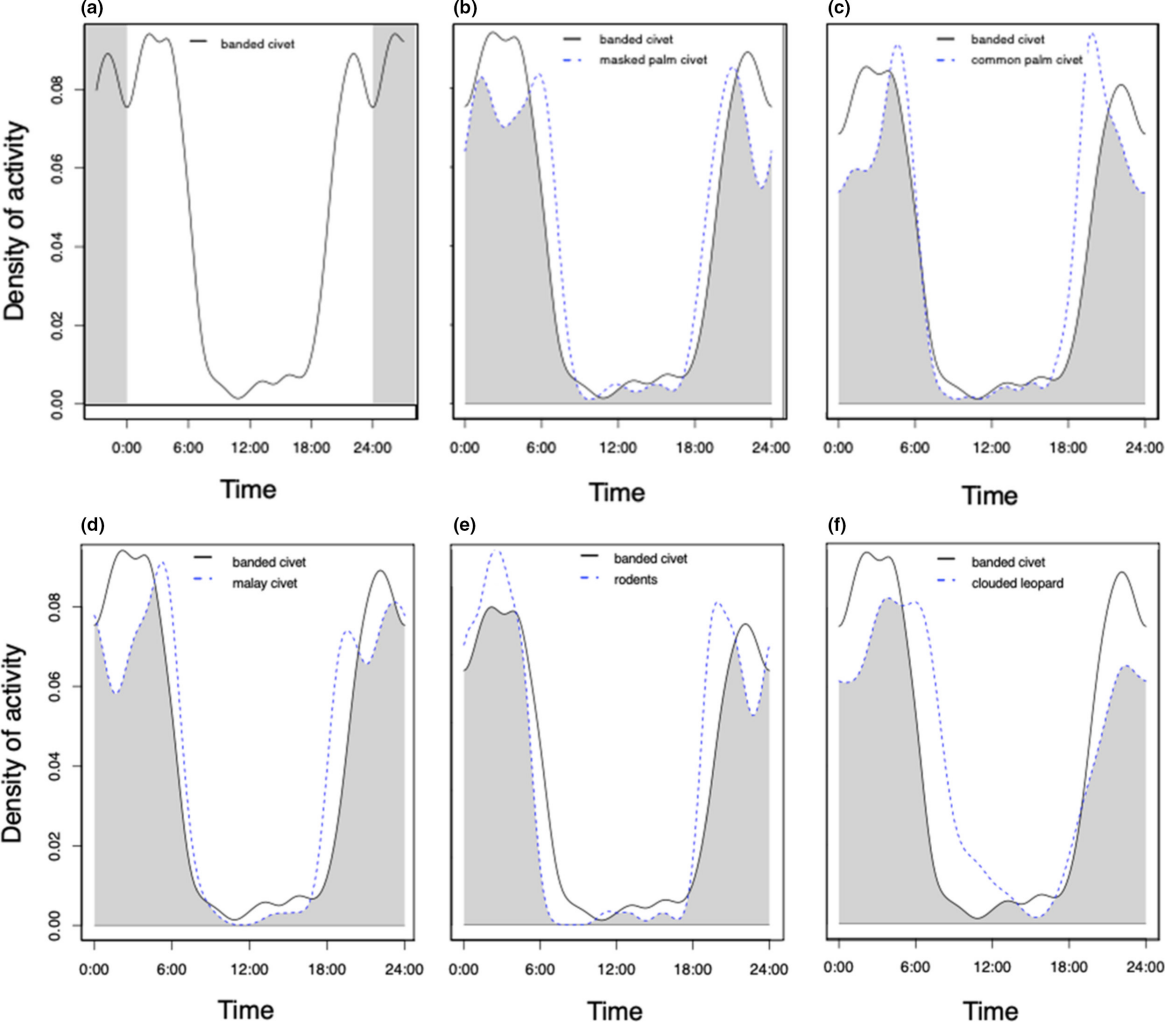
Activity patterns and temporal overlap of banded civet (a) and potential competitors (other civets, b–d), prey (rodents of the Muridae family, e), and predator (clouded leopard, f)

**FIGURE 6:**
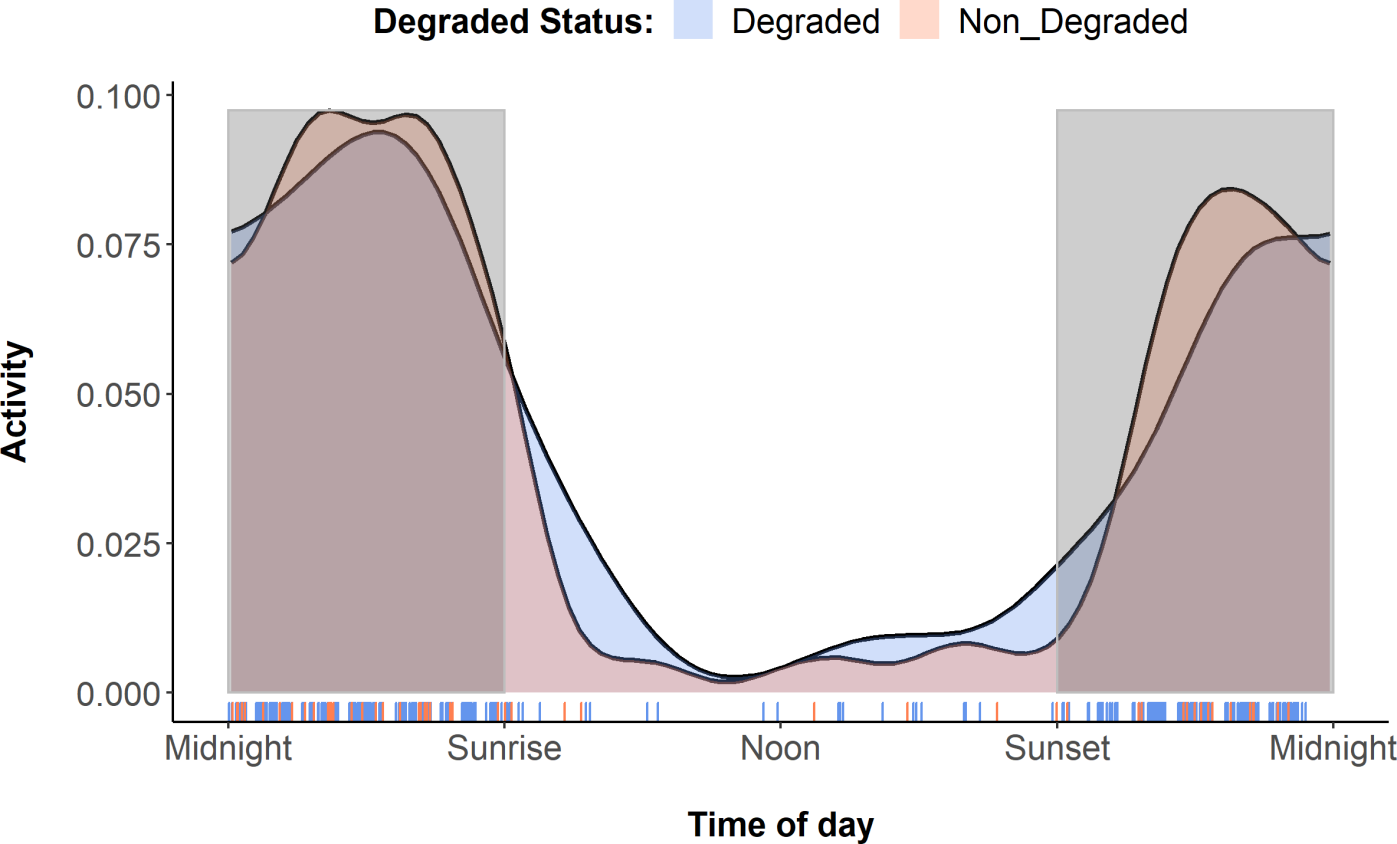
Variation in the activity patterns of banded civet. Comparing banded civet activity patterns across degraded vs pristine land cover. *Note we excluded landscapes with <4 captures

The activity patterns showed banded civets are strictly nocturnal and not crepuscular as there were no distinctive activity peaks around dawn and dusk (Figure 5a). There was extremely high temporal overlap between banded civet and the masked palm civet (Dhat_4_ = 0.86), malay civet (Dhat_4_ = 0.85) and common palm civet (Dhat_4_ = 0.85), as well as with likely prey (rodents in the Muridae family) (Dhat_4_ = 0.85) and a potential predator, the clouded leopard (*Neofelis nebulosa*; Dhat_4_ = 0.81). We found no significant change in the banded civets’ activity patterns between high and low forest edges, forest integrity or human footprint values (Wald test statistic = 0.16, *p* = .68; *W* = 0.07, *p* = .79; *W* = 0.38, *p* = .54, respectively; Figure 6).

## 4 DISCUSSION

Our synthesis strongly supports banded civets being a nocturnal forest‐specialist that avoids degraded areas and therefore is unlikely to have a high overlap with humans in the wild. Prior observations of banded civets in edges and degraded forests do not appear to reflect general habitat associations of the species we determined using larger datasets and more robust analyses. Previous research has found that other mammals become more nocturnal around disturbance (Gaynor et al., 2018) however we found change in the banded civets’ activity pattern. Other species of Asian civets are often found in degraded forest edges and even consume crops or rodent pests, such as the Malay civet (*Viverra tangalunga*), common palm civet (*Paradoxurus hermaphroditus*), and masked palm civet (*Paguma lavarta*) (Dehaudt et al., 2022; Nakashima et al., 2013; Parrish et al., 2008), while we noted positive associations between banded civets and forest cover and distance from rivers and a negative association with human population (Figure 4). This suggests riparian habitats are not key habitats, and since rivers are often used by humans, this also reduces the likelihood of human‐civet interactions. Taken together, our findings suggest banded civets prefer more intact forest landscapes and there is no evidence to suggest “mesopredator release” (higher abundances in degraded areas), and therefore banded civets may arguably be considered a low‐risk vector of zoonotic diseases in natural settings.

### 4.1 Zoonotic disease (ZD) risks from civets

There are numerous other pathways where banded civets may contribute to ZD aside from overlapping with humans in natural settings, especially if banded civets may be targeted for trade (Morand & Lajaunie, 2021). The most recent global ZD of SARS‐CoV‐2 (COVID‐19) most likely originated from bats (Zhu et al., 2020) and may have been transmitted to humans via an intermediary host, such as civets, through close contact in wildlife markets (Shereen et al., 2020). This is supported by evidence about the emergence of a SARS‐CoV‐1 transmission pathway involving civets, and SARS‐CoV‐2 infecting other mesopredators such as minks (*Neovison vison*) which then transmitted the disease to humans in Europe (Frutos & Devaux, 2020). Further, banded civets may act as a link or reservoir in the transmission of ZDs among other related sympatric civet species in the wild or captivity, and we noted high temporal activity overlap between the banded civet with other civet species showing more human‐commensal behaviors (Parrish et al., 2008). Efforts to reduce the chances of ZDs emergence from civets include regulations on capture and trade of all civets, deterring civet consumption or keeping civets as pets, and discouraging live animal markets. There have also been calls to increase the local and international protection (e.g., CITES) for all civets regardless of their conservation threat status (Frutos & Devaux, 2020).

### 4.2 Banded civet conservation

We suggest the species IUCN‐RL threat status be re‐evaluated in light of the three developments since the last assessment. First, ongoing deforestation in the region has reduced the species Area of Habitat (AOH) (GWF, 2021). As of 2015, just 34% of the prior IUCN‐RL Extent of Occurrence (EOO) remains forested and could be considered AOH and only 11.7% of the EOO is protected. These results suggest a rapid contraction in the species’ AOH given the last IUCN‐RL assessment was published in 2015 and would have used remotely sensed habitat layers from the mid‐late 2000s to estimate EOO in remaining areas with tree cover (Ross et al., 2015). This is particular problem because older tree cover layers overestimated forest cover in Southeast Asia since they could not differentiate forest from the mature tree plantations (oil palm, rubber, acacia) that proliferate in the region (Hansen et al., 2013; Luskin & Potts, 2011). Second, the habitat associations we observed (avoidance of forest degradation) infer the true occupancy within the AOH may be limited to the largest and most intact forest. However, the vast majority of remaining forests in the region are within 1 km of an edge and suffer various other forms of degradation (Grantham et al., 2020; Haddad et al., 2015). The prior IUCN‐RL assessment may have overestimated the AOH if they assumed relatively homogeneous occupancy in remaining forests, while in fact, the species only shows high occupancy in relatively large forest interiors. A final reason for upgrading the IUCN‐RL threat status is to match similar species in the region for which there was previously more data available. For reference, the more charismatic binturong (*Arctictis binturong*) is listed as Vulnerable, and there is no evidence the banded civet faces lower threats since both species have a similar range, habitat associations, and behavior (forest‐dependent, semi‐arboreal, nocturnal) (Wilcox et al., 2016).

### 4.3 Future research directions

There are several remaining gaps in our knowledge of the banded civet's ecology, including details about its diet and interactions with other civets. For instance, it is not clear whether they consume fruits, as do many other omnivorous civets that are considered important seed dispersers. There is little understanding of the factors regulating the species’ populations or of their role in regulating the population of smaller prey species. Likewise, their movement, home‐range size, territoriality, and their mating behavior or breeding in the wild have not been studied. Finally, there are enduring community ecology questions about the coexistence and niche complementarity of the numerous sympatric Asian civets that exhibit many similarities in diet, habitat associations, and behavior.

## CONFLICT OF INTEREST

The authors declare that they have no conflict of interest.

## AUTHOR CONTRIBUTIONS

**Ashlea Dunn:**Formal analysis (equal); Investigation (equal); Project administration (equal); Writing – original draft (equal); Writing – review & editing (equal). **Zachary Amir:** Conceptualization (equal); Methodology (equal); Software (equal); Supervision (equal); Validation (equal); Visualization (equal); Writing – review & editing (equal). **Henri Decoeur:** Methodology (equal); Project administration (equal); Writing – original draft (equal); Writing – review & editing (equal). **Bastien Dehaudt:** Methodology (equal); Writing – review & editing (equal). **Ilyas Nursamsi:** Formal analysis (equal); Methodology (equal); Writing – review & editing (equal). **Calebe Mendes:** Data curation (equal); Formal analysis (equal); Methodology (equal); Writing – review & editing (equal). **Jonathan H. Moore:** Data curation (equal); Methodology (equal); Writing – review & editing (equal). **Pablo Jose Negret:** Methodology (equal); Writing – review & editing (equal). **Adia Sovie:** Methodology (equal); Writing – review & editing (equal). **Matthew Scott Luskin:** Conceptualization (lead); Data curation (equal); Formal analysis (equal); Investigation (equal); Methodology (equal); Project administration (equal); Resources (lead); Software (equal); Supervision (lead); Validation (equal); Visualization (equal); Writing – original draft (equal); Writing – review & editing (lead).

## Supporting information

Appendix S1

## Data Availability

Data and R code are available via the Dryad Digital Repository https://doi.org/10.5061/dryad.8cz8w9gs1.
