# Supplementary material for: The ecology of the banded civet (Hemigalus derbyanus) in Southeast Asia with implications for mesopredator release, zoonotic diseases, and conservation
Source: Ecol Evol. 2022 Apr 28;12(5):e8852. doi: 10.1002/ece3.8852 (PMC9047978; doi:10.1002/ece3.8852)
Supplement: Supplementary file 1 — Appendix S1 [file ECE3-12-e8852-s001.docx]

# SUPPLEMENTARY MATERIALS

**Additional species descriptions**

In captivity, the records state females are noticeably pregnant 3 weeks before birth (Louwman 1970). Additional observations in captivity have noted specific calls including; hissing, spitting, whining and growling (Phillipps and Phillipps 2018). The species is listed as Appendices II in CITES which requires a valid export permit in the trade of *H. derbyanus* (<http://www.cites.org/eng/app/appendices.php>).

**Table S1**. **Landscape description and survey effort for new camera trapping.** Trap nights were estimated between the first and last photos taken for each collected camera. MCP refers to the minimum convex polygon around the camera traps. To account for variation in deployment scale and spacing, we resampled all data by grouping cameras into 1-km apothem hexagonal units (3.45 km² cells). Therefore, the rows in the capture histories all represent the same sampling area, and because some cells had more than one camera, we included trapping effort per cell per sampling window as a covariate of detection.

| Survey | Annual rainfall | | Cameras collected | Effort (Trap nights) | Duration | Elevation  (Mean ± SD) | Elevation range | MCP | Camera spacing |
| --- | --- | --- | --- | --- | --- | --- | --- | --- | --- |
| THAILAND |  | |  |  |  |  |  |  |  |
| Khao Chong area of Khao Ban Tat 2018 | 2014.28 | | 76 | 3957 | 2018-02-01 – 2018-04-30 | 524.59 ± 270.92 | 103 – 1234 | 59.01 | 467.95 |
| Khao Yai 2019  (outside the banded civet range) | 1119.49 | | 61 | 3553 | 2019-07-01 – 2019-09-25 | 769.64 ± 38.56 | 582 – 816 | 22.54 | 464.42 |
| SUMATRA |  | |  |  |  |  |  |  |  |
| Gunung Leuser 2014 | 2828.00 | | 69 | 3401 | 2013-12-18 – 2014-05-22 | 316.03 ± 250.38 | 25 – 888 | 516.15 | 1275.27 |
| Kerinci Seblat 2014 | 2406.94 | | 98 | 5356 | 2014-02-10 – 2014-10-04 | 594.03 ± 194.46 | 252 – 1154 | 813.69 | 1169.04 |
| Bukit Barisan Selatan 2014 | 2987.80 | | 79 | 5750 | 2014-06-15 – 2014-09-20 | 369.75 ± 184.97 | 116 – 935 | 473.58 | 1139.96 |
| MALAYSIAN BORNEO |  | |  |  |  |  |  |  |  |
| Danum Valley 2019  (Sabah) | 2182.68 | | 22 | 1292 | 2019-05-24 – 2019-09-26 | 256.73 ± 102.02 | 184 – 567 | 8.31 | 520.76 |
| Danum Valley 2018  (Sabah) | 2182.85 | | 27 | 1849 | 2018-07-12 – 2018-10-30 | 249.63 ± 53.23 | 175 – 381 | 15.95 | 614.15 |
| Lambir Hills 2017  (Sarawak | 3078.82 | | 67 | 2406 | 2017-05-23 – 2017-07-07 | 164.80 ± 65.31 | 60.31 – 421.44 | 22.06 | 459.95 |
| PENINSULAR MALAYSIA | |  |  |  |  |  |  |  |  |
| Pasoh 2013 | 2081.40 | | 58 | 1399 | 2013-05-29 – 2014-02-12 | 297.09 ± 160.10 | 98 – 674 | 133.53 | 1316.26 |
| Pasoh 2014 | 2079.16 | | 57 | 1314 | 2014-05-13 – 2014-08-01 | 303.14 ± 160.04 | 98 – 674 | 134.62 | 1321.48 |
| Pasoh 2015 | 2079.78 | | 59 | 1670 | 2015-05-07 – 2015-09-04 | 301.15 ± 158.15 | 98 – 674 | 134.62 | 1317.78 |
| Pasoh 2016 | 2086.38 | | 42 | 1305 | 2017-05-17 – 2017-08-29 | 308.98 ± 156.05 | 103 – 674 | 122.63 | 1416.43 |
| Ulu Muda 2015a | 2057.03 | | 76 | 4242 | 2014-11-01 – 2015-01-30 | 278.58 ± 128.68 | 117 – 628 | 68.98 | 938.65 |
| Ulu Muda 2015b | 2063.01 | | 112 | 4446 | 2015-01-31 – 2015-05-01 | 295.77 ± 139.83 | 117-843 | 113.61 | 731.56 |
| Ulu Muda 2015c | 2080.90 | | 52 | 3582 | 2015-05-02 – 2015-07-31 | 325.38 ± 166.15 | 141-843 | 115.53 | 1227.86 |
| Ulu Muda 2015d | 2078.17 | | 48 | 2862 | 2015-08-01 – 2015-10-30 | 328.92 ± 165.27 | 123-843 | 104.01 | 1237.85 |
| Ulu Muda 2016a | 2065.89 | | 73 | 2220 | 2015-10-31 – 2016-01-29 | 313.62 ± 145.27 | 117-748 | 103.17 | 794.92 |
| Ulu Muda 2016b | 2054.55 | | 60 | 2899 | 2016-01-30 – 2016-04-29 | 285.45 ± 135.12 | 117-628 | 66.96 | 958.39 |
| Ulu Muda 2016c | 2060.54 | | 46 | 2746 | 2016-04-30 – 2016-07-22 | 301.30 ± 138.91 | 117-628 | 65.72 | 974.47 |
| SINGAPORE |  | |  |  |  |  |  |  |  |
| Singapore 2019 | 2283.97 | | 36 | 2359 | 2018-12-26 – 2019-03-17 | 41.44 ± 22.18 | 0 – 83 | 162.35 | 261.70 |

**Table S2. Description and sources of spatial data used in the studies analysis**

| Covariate Type | Covariate source, description or calculation | Year | Resolution | | Source hosting the layer |
| --- | --- | --- | --- | --- | --- |
| Forest cover | Natural tree cover (excluding plantations) | 2015 | | 1m | (CRISP), National University of Singapore^1^ |
| Forest edge | Distance to forest edge | 2015 | | 1m | (CRISP), National University of Singapore^1^ |
| Oil palm | Industrial plantations only, likely captures a minority of total oil palm | 2015 | | 1m | (CRISP), National University of Singapore^1^ |
| Degraded forest | Combined land cover of oil palm, lowland mosaics, lowland open ground, and regrowth/plantations | 2015 | | 1m | (CRISP), National University of Singapore^1^ |
| Forest integrity | Forest Landscape Integrity Index^2^ capturing direct and indirect pressures on forest. | 2020 | | 300m | <https://www.forestlandscapeintegrity.com> |
| Elevation | SRTM Digital Elevation | 2020 | | 30m | https://dwtkns.com/srtm30m/ |
| Land cover | MODIS classification system | 2015 | | 250m | (CRISP), National University of Singapore^1^ |
| Human density | Human settlements and population | 2015 | | 250m | https://ghsl.jrc.ec.europa.eu/ |
| Settlements | Overpass Turbo - OSM | 2020 | | - | http://overpass-turbo.eu/ |
| Roads | OpenStreetMap | 2020 | | - | https://www.openstreetmap.org |
| Rivers | GRIN - Global River Network | 2017 | | - | https://www.metis.upmc.fr/en/node/375 |
| Human footprint | Human Footprint Index^3^ showing cumulative human pressures from direct and indirect sources. | 2009 | | 1km | https://sedac.ciesin.columbia.edu/ |
| Country boundaries | Country Boundaries | 2020 | | - | https://gadm.org/download_country_v3.html |
| Night lights | DMSP-OLS Night-time Lights | 2013 | | 1km | https://eogdata.mines.edu/dmsp/ |
| Forest Loss | Forest Change 2000–2019 | 2019 | | 30m | Global forest watch^4^ |
| Protected areas | IUCN and UNEP-WCMC, The World Database on Protected Areas (WDPA) | 2017 | | shape | www.protectedplanet.net |
| ^1^ (Miettinen et al. 2016)  ^2^ (Grantham et al. 2020)  ^3^ (Venter et al. 2016)  ^4^<https://earthenginepartners.appspot.com/science-2013-global-forest/download_v1.7.html> | | | | | |

**Table S3: Range, habitat availability and naïve occupancy of the banded civet per country.** EOO refers to the extent of occurrence, which we calculated as the total area within the IUCN-RL range in each region (km²). We updated the EOO based on the forested area in 2015 remaining within the IUCN EOO (Miettinen et al. 2016), which may be interpreted more correctly as the remaining habitat available. Protected areas were taken from Protected Planet database (IUCN 2010).

| **Country** | **IUCN-RL EOO**  **(km^2^)** | **IUCN-RL EOO that remains forested (km^2^)** | **IUCN-RL EOO that remains forested (%)** | **IUCN-RL EOO that remains forested and is protected (%)** |
| --- | --- | --- | --- | --- |
| Brunei | 5815 | 3217 | 55.3 | 17 |
| Indonesia | 967708 | 319278 | 33 | 3.3 |
| Malaysia | 328467 | 134182 | 40.9 | 10.5 |
| Myanmar | 57 | 43 | 74.9 | 2.2 |
| Thailand | 57267 | 12022 | 21 | 16.3 |
| *Totals* | *1359592* | *468745* | *34.5* | *7.9* |

**Table S4. Landscape-level naïve occupancy of the banded civet by country.** The main text presents results by region.

| **Location** | **Landscapes surveyed** | **Landscapes with detections** | **Naïve occupancy** | **Camera stations** | **Effort trap nights** | **Independent detections** | **RAI** |
| --- | --- | --- | --- | --- | --- | --- | --- |
| Brunei | 0 | 0 | 0 | 0 | 0 | 0 | 0 |
| Indonesia | 8 | 5 | 0.63 | 1734 | 126038 | 163 | 0.129 |
| Malaysia | 30 | 21 | 0.7 | 3018 | 176710 | 768 | 0.435 |
| Myanmar | 0 | 0 | 0 | 0 | 0 | 0 | 0 |
| Thailand | 3 | 3 | 1 | 235 | 26165 | 55 | 0.21 |
| **Southeast Asian total** | **41** | **29** | **0.71** | **4987** | **328913** | **986** | **0.3** |

**Table S5. Landscape surveys and their naïve occupancy and RAI.**

|  |  |  |  |  |  |  |
| --- | --- | --- | --- | --- | --- | --- |
| **Region** | **Landscape** | **Year** | **Naïve Occupancy** | **Independent Captures** | **Effort (trap nights)** | **RAI - Captures per 100 trap nights** |
| Sumatra | BBS | 2014 | 0.1923077 | 33 | 5750 | 0.573913043 |
| Borneo | Danum Valley | 2019 | 0.4615385 | 18 | 1292 | 1.393188854 |
| Borneo | Danum Valley | 2018 | 0.0526316 | 1 | 1849 | 0.054083288 |
| Sumatra | Kerinci Seblat | 2014 | 0.0178571 | 3 | 5356 | 0.056011949 |
| Thailand | Khao Chong | 2018 | 0.0227273 | 1 | 3957 | 0.02527167 |
| Sarawak | Lambir Hills | 2017 | 0.0967742 | 6 | 2406 | 0.249376559 |
| Sumatra | Gunung Leuser | 2014 | 0.1449275 | 17 | 3401 | 0.499852984 |
| Peninsular Malaysia | Pasoh | 2013 | 0.0862069 | 5 | 1399 | 0.357398142 |
| Peninsular Malaysia | Pasoh | 2014 | 0.0526316 | 4 | 1314 | 0.304414003 |
| Peninsular Malaysia | Pasoh | 2015 | 0.0508475 | 4 | 1670 | 0.239520958 |
| Peninsular Malaysia | Pasoh | 2017 | 0.0238095 | 1 | 1305 | 0.076628352 |
| Peninsular Malaysia | Ulu Muda a | 2015 | 0.4179104 | 90 | 4242 | 2.121640736 |
| Peninsular Malaysia | Ulu Muda b | 2015 | 0.3139535 | 80 | 4446 | 1.79937022 |
| Peninsular Malaysia | Ulu Muda c | 2015 | 0.2307692 | 20 | 3582 | 0.558347292 |
| Peninsular Malaysia | Ulu Muda d | 2015 | 0.125 | 13 | 2862 | 0.454227813 |
| Peninsular Malaysia | Ulu Muda a | 2016 | 0.1525424 | 17 | 2220 | 0.765765766 |
| Peninsular Malaysia | Ulu Muda b | 2016 | 0.2 | 28 | 2899 | 0.965850293 |
| Peninsular Malaysia | Ulu Muda c | 2016 | 0.3902439 | 30 | 2746 | 1.092498179 |
| * We also sampled Singapore and Khao Yai in Thailand but the species was not detected. | | | | | | |


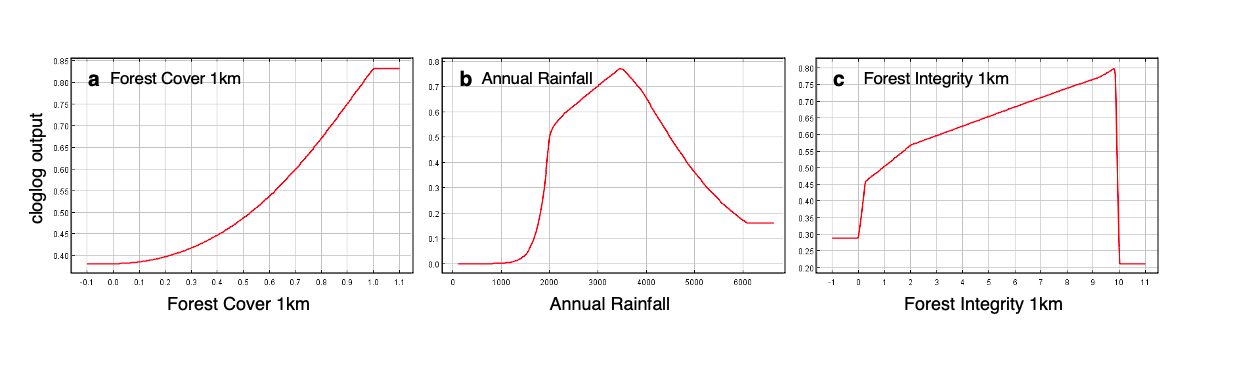


**Figure S1**: Response curves from MaxEnt of the important variables to the species probability of presence: Response curve for; (a) Forest cover, (b) Rainfall, (c) Forest integrity.
